# ^1^H-Nuclear magnetic resonance-based metabolomic analysis of brain in mice with nicotine treatment

**DOI:** 10.1186/1471-2202-15-32

**Published:** 2014-02-22

**Authors:** Hongyu Li, Bo Chen, Xue Shao, Zhengtao Hu, Yi Deng, Ruiming Zhu, Yan Li, Baolai Zhang, Jing Hou, Changman Du, Qian Zhao, Dengqi Fu, Qian Bu, Yinglan Zhao, Xiaobo Cen

**Affiliations:** 1 National Chengdu Center for Safety Evaluation of Drugs, State Key Lab of Biotherapy, West China Hospital, Sichuan University, 28# Gaopeng Avenue, High Technological Development Zone, Chengdu 610041, China

**Keywords:** Metabolomics, Nicotine, Metabolite, NMR, Place preference

## Abstract

**Background:**

Nicotine is rapidly absorbed from cigarette smoke and therefore induces a number of chronic illnesses with the widespread use of tobacco products. Studies have shown a few cerebral metabolites modified by nicotine; however, endogenous metabolic profiling in brain has not been well explored.

**Results:**

H NMR-based on metabonomics was applied to investigate the endogenous metabolic profiling of brain hippocampus, nucleus acumens (NAc), prefrontal cortex (PFC) and striatum. We found that nicotine significantly increased CPP in mice, and some specific cerebral metabolites differentially changed in nicotine-treated mice. These modified metabolites included glutamate, acetylcholine, tryptamine, glucose, lactate, creatine, 3-hydroxybutyrate and nicotinamide-adenine dinucleotide (NAD), which was closely associated with neurotransmitter and energy source. Additionally, glutathione and taurine in hippocampus and striatum, phosphocholine in PFC and glycerol in NAc were significantly modified by nicotine, implying the dysregulation of anti-oxidative stress response and membrane metabolism.

**Conclusions:**

Nicotine induces significant metabonomic alterations in brain, which are involved in neurotransmitter disturbance, energy metabolism dysregulation, anti-oxidation and membrane function disruptions, as well as amino acid metabolism imbalance. These findings provide a new insight into rewarding effects of nicotine and the underlying mechanism.

## Background

Nicotine is considered as the major addictive component of tobacco smoke, resulting in addiction in humans [1,2]. According to the World Health Organization, nicotine addiction is a global health problem affecting one-third of the population [3]. Some highly addicted smokers attempt to quit smoking; however, only 3–5% are successful without the use of nicotine replacement therapies, and no more than one-third are successful with them [4]. It is known that the addiction behavior caused by nicotine is involved with rewarding effects of nicotine. Therefore, much effort has been carried out to explore the mechanism of rewarding effects of nicotine. Nicotine stimulates the brain areas containing nicotinic acetylcholine receptor (nAChR) subunits, leading to the release of a variety of neurotransmitters [5]. Ventral tegmental area (VTA) of the midbrain, which projects to the prefrontal cortex (PFC), limbic, striatal structures, and nucleus accumbens (NAc), contributes to nicotine intake and reinforcement [6,7]. However, these studies have only focused on certain AChR subunits and/or neurotransmitters, which have already been known to participate in rewarding effects of nicotine, lacking endogenous metabolic profiling in a global view. We wondered if the rewarding effects of nicotine on the brain were related to some endogenous metabolites. Therefore, we tried to detect changes of metabolites in numerous brain regions of mice after nicotine treatment.

Currently, metabonomics, as a promising approach, has been widely applied in neuropsychiatric research field, such as drug addiction, motor neuron disease, schizophrenia and Parkinson’s disease [8,9]. Additionally, multivariate analysis of metabolic profiles reveal that smoking is associated with plasmalogen-deficiency disorders [10]. Unlike genomics, transcriptomics and proteomics which indicate what may happen, metabonomics measure all metabolites, which determine the state of the pathophysiology of organism and have the potential to identify related biomarkers. Nuclear magnetic resonance (NMR) is one of the analytical methods in metabolomics research and has been widely applied to explore multiple metabolic changes in brain tissues [11,12]. For example, ^1^H NMR-based metabonomics shows the neurotransmitter disturbance in brain NAc and striatum of cocaine-treated rats [13]. Neurotransmitter and purine metabolic pathway are likely affected by addictive substances, including morphine and cocaine [14]. Therefore, ^1^H NMR-based metabonomics was applied to explore the changes of metabolites in brain after nicotine treatment in our study.

Conditioned place preference (CPP) is a naturalistic model of addictive behavior. CPP measures an animal’s preference for a particular place in its environment, which becomes closely contacted with a rewarding stimulus and assumes the rewarding effects of the stimulus [15]. CPP had been selected to explore the rewarding effects of nicotine in many studies. In our study, CPP was chosen to explore nicotine-induced rewarding effects [16,17].

In the present study, we developed CPP induced by nicotine in C57BL/6 J mice and applied ^1^H NMR-based metabonomics coupled with principal component analysis (PCA), partial least squares (PLS) and orthogonal signal correction (OSC) to examine the changes of metabolic profile in the brain hippocampus, NAc, PFC and striatum. We found that nicotine can result in neurotransmitter disturbance, oxidative stress alteration, mitochondria dysregulation, membrane disruption, energy metabolism imbalance, and amino acid disorder. Our findings provide a new insight into the neurobiological and biochemical changes associated with rewarding effects of nicotine.

## Methods

### Drugs

Nicotine hydrogen tartrate (doses expressed as base) was purchased from Sigma (Beijing, China). Nicotine was dissolved in 0.9% sterile saline before use.

### Animals and administration

C57BL/6 J mice (16 male, 8-12 weeks old) were kept in clear plastic cages with five per cage at under a 12/12 h light–dark cycle in room temperature (21 ± 5°C) with a relative humidity of 55 ± 15% and an air change rate of 8–10 changes/hour, and given food and water. The animals were acclimatized for 7 days before experiment. This study was carried out in accordance with the guidelines established by the Association for Assessment and Accreditation of Laboratory Animal Care. The protocols were approved by the Committee on the Ethics of Animal Experiments of National Chengdu Center for Safety Evaluation of Drugs, West China Hospital of Sichuan University (Protocol number IACUC-S200909-P001). All surgery was performed under sodium pentobarbital anesthesia, and all efforts were made to minimize suffering.

### Nicotine CPP

Place conditioning studies were conducted using a shuttle box which was composed of three distinct chambers including two large conditioning chambers and a small central start chamber. During pretest phase, on day 0, mice were placed in the central chamber, and allowed to move the apparatus for 15 min. The time spent in each chamber was recorded. Mice with a chamber bias greater than 75% were dropped from studies and unbiased mice were randomly assigned to two groups including control group (0.9% sodium chloride only) and nicotine group (0.5 mg/kg nicotine). Nicotine and saline were administrated by subcutaneous injection (s.p). On days 1–3, in nicotine group, mice were given one injection of nicotine (0.5 mg/kg) on the non-preferred chamber by closing the removable wall for a period of 15 min and one injection of saline on the preferred chamber each day for a period of 15 min (one in the morning and one in the afternoon 4 hours later in a counterbalanced manner); moreover, in control group, nicotine was replaced by saline and the rest procedures were as described above. During posttest phase, on the fifth day, CPP was tested. Each mouse was allowed to move freely between all the chambers for 15 min and the time spent in each chambers was measured. Data were expressed as the changed time in posttest minus the changed time in pretest, and the changed time is time spent in the preferred chamber minus time spent in the non-preferred chamber. And each group was presented as mean ± SD. One-way analysis of variance (ANOVA) followed by Tukey post hoc test was used to determine statistical significance.

### Preparation of brain extracts

Mice were sacrificed by cervical dislocation within 30 minutes after the end of CPP posttest. Specimens of bilateral brain hippocampus, NAc, PFC and striatum (~30-100 mg) were dissected immediately, snap-frozen in liquid nitrogen, and stored at -80°C until NMR spectroscopic analysis. The preparation of the brain samples was based on the previous studies [18,19]. The frozen tissue was added into 0.5 ml chloroform and homogenized at 4°C. The supernatant was preserved to lyophilize for about 36 hours after centrifugation at 10, 000 g for 10 min at 4°C. Dried extracts after lyophilization were added into 480 μl D_2_O, including 20 μl0.25 mM sodium (3-trimethylsilyl)-2, 2, 3, and 3-tetradeuteriopropionate (TSP), and then transferred into 5 mm NMR tubes for ^1^H NMR detection.

### Solution ^1^H NMR Spectroscopy

^1^H NMR spectral data were acquired on a Bruker-Av II600 MHz spectrometer(Bruker Co, Rheinstetten, Germany)at 300 K.A standard (1D) Carr-Purcell-Meiboom-Gill (CPMG) pulse sequence was used to acquire a one-dimensional spectrum and suppress the water signal with a relaxation delay of 5 s. Typically, 64 free induction decays (FIDs) were collected into 64 k data points over a spectral width of 8992.8 Hz with a relaxation delay of 5 s and 0.91 s to total acquisition time. The FIDs were weighted by an exponential function with a 0.3-Hz line-broadening factor prior to Fourier transformation.

### Data reduction and pattern recognition analysis

All NMR spectra were automatically reduced to 218 segments, each with a 0.04 ppm width for a spectral window ranging from 9.5 to 0.2 ppm using MestRe-c 2.3 software (http://qobrue.usc.es/jsgroup/MestRe-c). The area for each segmented region was calculated. The segments of δ 4.6-5.1 ppm in spectra were removed to exclude the influence of the residual water resonance. The datasets were mean-centered prior to PCA, PLS and OSC analysis by the SIMCA package. PCA distinguished the characteristic variable (metabolic signals) or the outlier from the group by statistical method. Partial least squares (PLS), one of the most popular supervised pattern recognition (PR) methods, was served as the regression extension of principal component analysis (PCA) which is an unsupervised method [20]. Orthogonal signal correction (OSC) can filter unwanted variation from near-infrared spectral data. In order to strengthen the performance of subsequent multivariate pattern-recognition analysis and reinforce the predictive ability of the model, OSC was applied to optimize the separation in NMR-based metabonomic studies [21]. The variable importance plot (VIP) values and corresponding loadings for PLS models using OSC transformed data set were applied to identify the variable contribution to the position of spectra that were altered after drug treatment. ^1^H NMR chemical shifts and assignments of endogenous metabolites were conducted according to the previous literatures and the Human Metabolome Database (http://www.hmdb.ca/). Above methods have been well recognized and widely used in metabonomics [22,23].

## Results

### Nicotine-induced CPP

**Figure 1:**
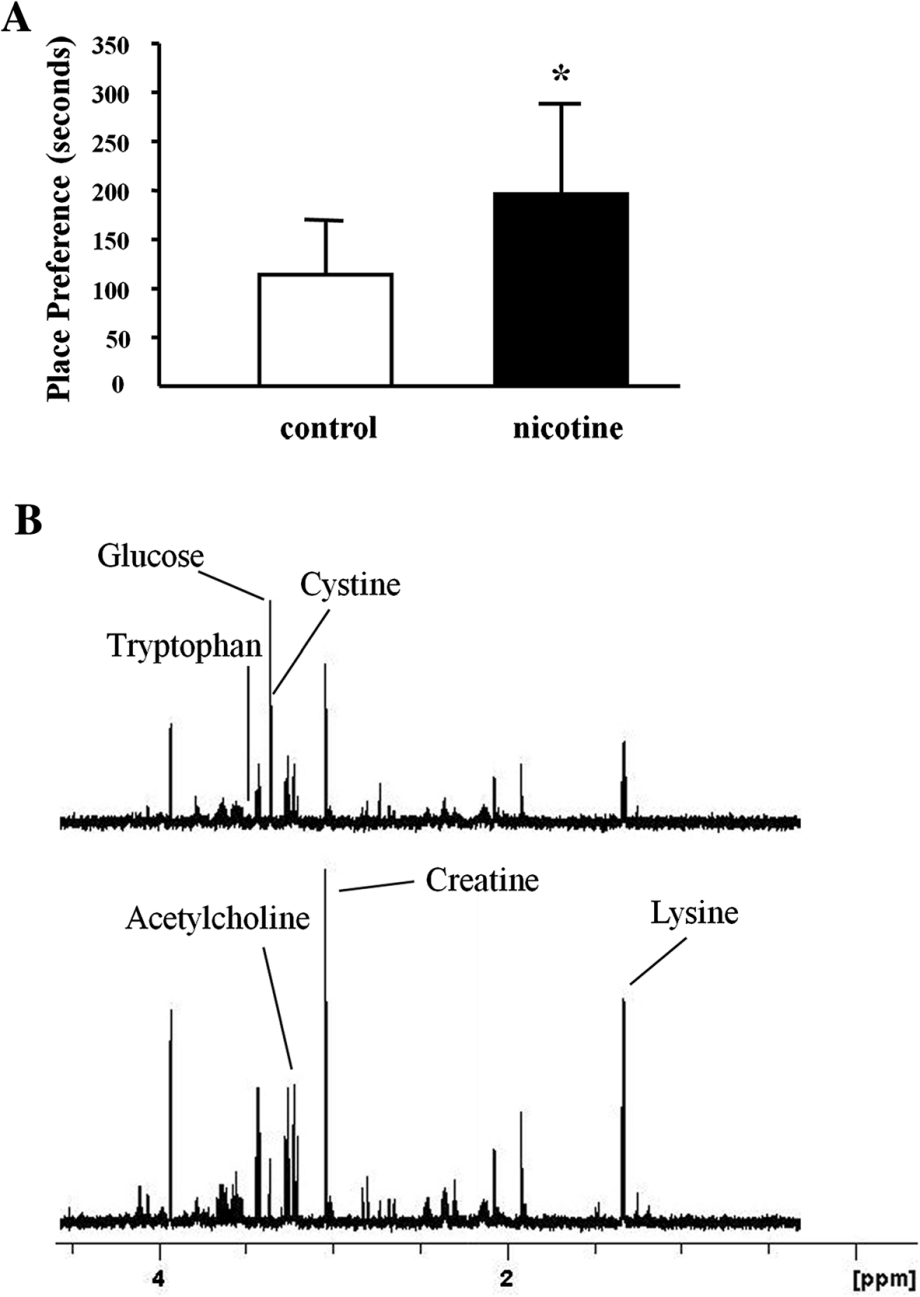
**Scores of nicotine-induced conditioned place preference (A) and 600 MHz CPMG **^**1**^**H NMR spectra of the striatum from mice (B). (A)** After repeated 0.5 mg/kg nicotine treatment, mice developed a significant preference for nicotine-paired side (n = 8, p < 0.05, F = 5.886); **(B)** the upper is control group; the bottom one is nicotine group.

Each group spent almost the same time on the initially non-preferred side, indicating that there were no basal differences between groups. The group receiving 0.5 mg/kg dose of nicotine spent significantly more time in the nicotine-paired chamber on the posttest day than the pretest day (P < 0.05)(Figure 1A). This result showed that injection of nicotine was sufficient to increase CPP in C57BL/6 J mice.

### NMR spectra and PLS-DA analysis

**Figure 2:**
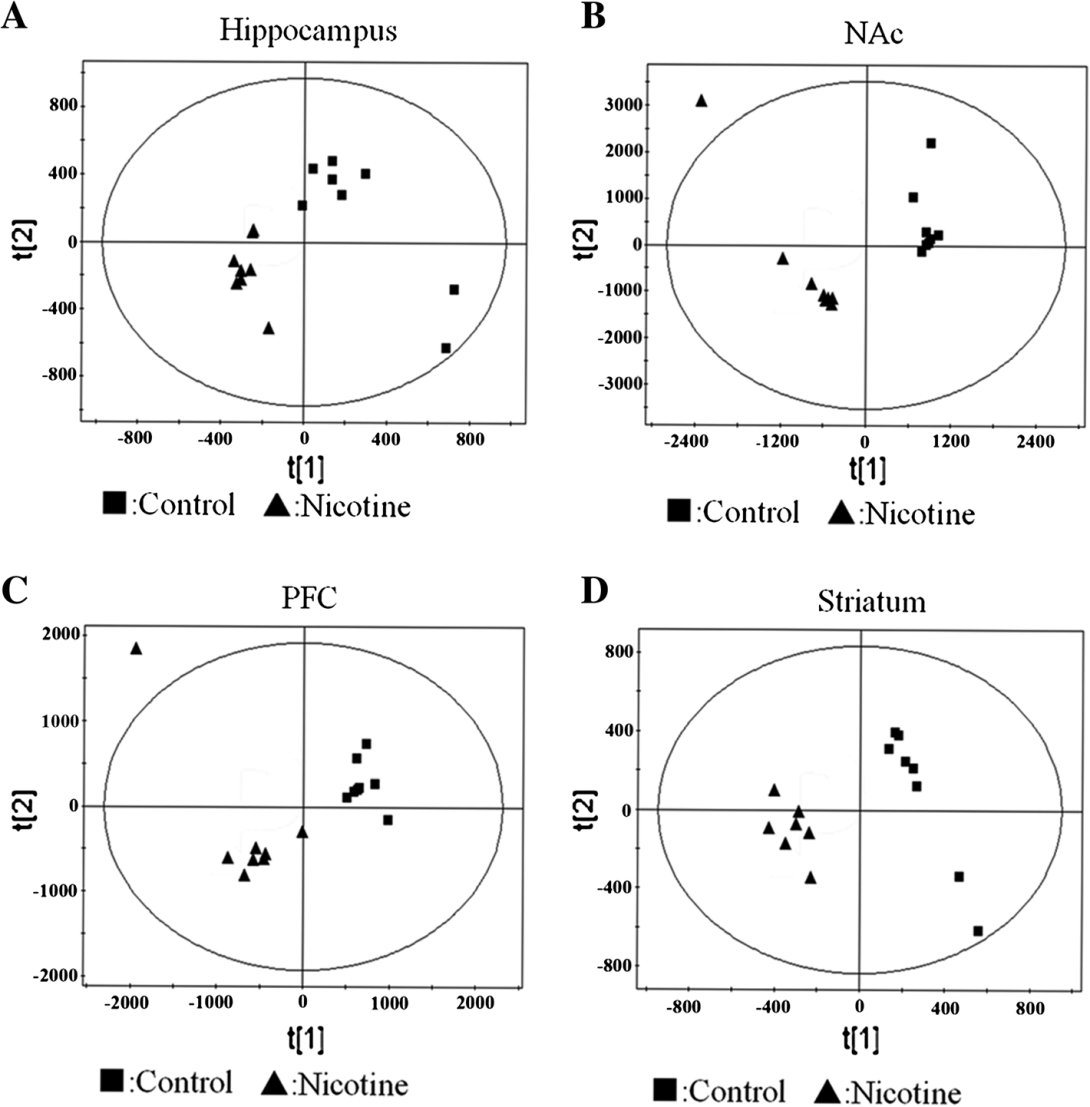
PLS scores plots and OSC-PLS scores plots of brain regions from mice: (A) hippocampus; (B) NAc; (C) PFC; (D) striatum.

Representative ^1^H NMR spectra of the water extracts of the striatum in control and nicotine groups were shown in Figure 1B with major metabolites in the integrate regions assigned. Visual inspection of ^1^H NMR spectra displayed distinctions between control and nicotine groups. To further investigate these distinctions, we utilized PCA, PLS and OSC to uncover the latent biochemical information from brain hippocampus, NAc, PFC and striatum. We firstly applied PCA as an unsupervised PR method to analyze the data sets of the ^1^H NMR spectra. Subsequently, we augmented this separation by using PLS, as a supervised PR method. However, aforementioned two PR methods displayed no clear separation in the brain NMR spectra for the first two principal components (PCs) in each treated groups. Then, PLS model following OSC was performed to further separate these treated groups. Each brain region of treated groups showed a significant differentiation in the PLS scores plots after application of OSC-PLS model (Figure 2).

**Figure 3:**
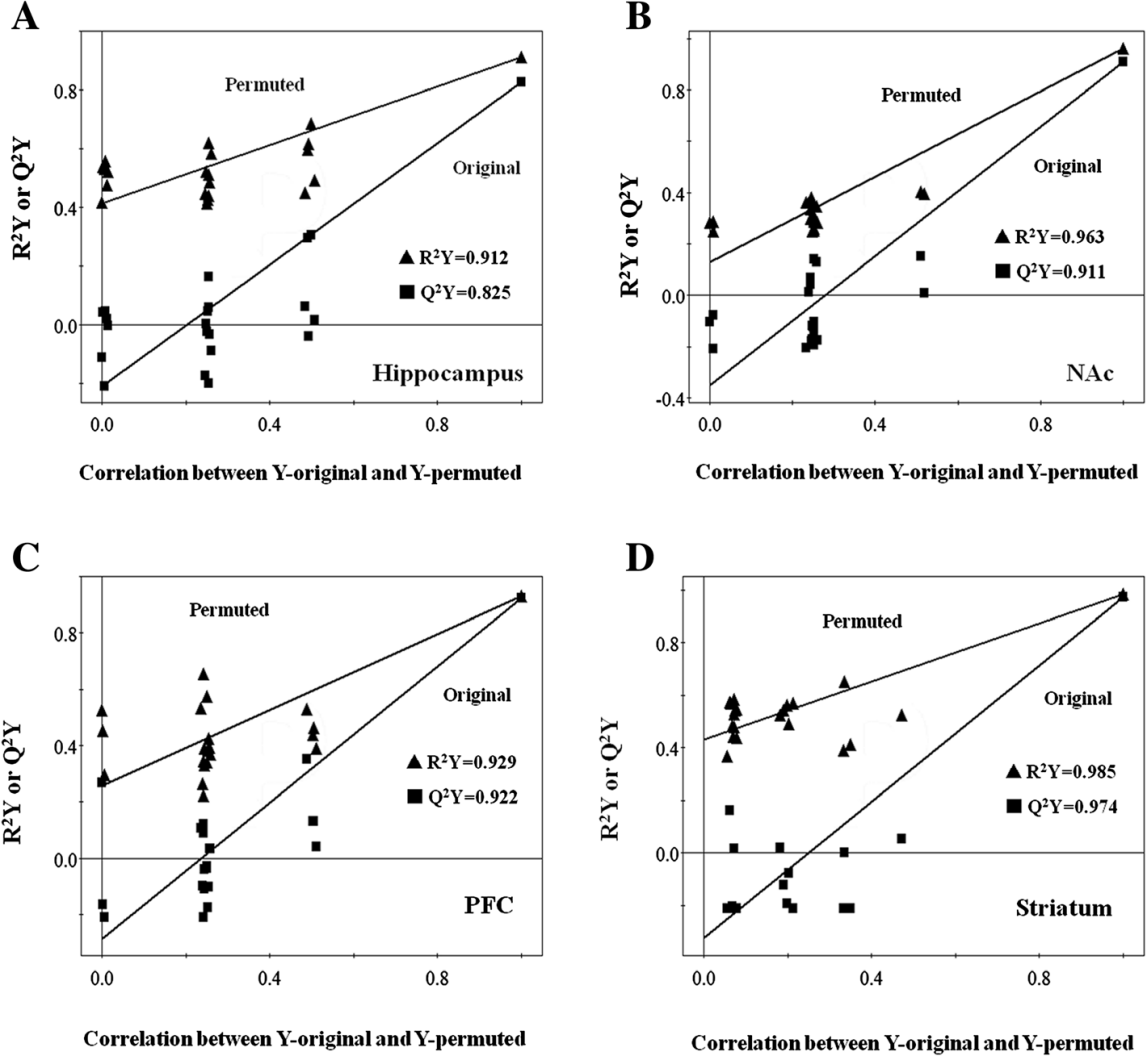
Validation plots of the PLS models after application of OSC: (A) hippocampus; (B) NAc; (C) PFC; (D) striatum.

In order to reach a credible conclusion, the OSC-PLS model was validated by a permutation method. The idea of this validation was to compare the goodness of fit (R^2^Y) and the predictability (Q^2^Y) of the original model based on data. R^2^Y was mathematically reproduced varies between 0 and 1, where 1 indicated a model with a perfect fit. Q^2^Y values of >0.5 and >0.9 implied good and excellent predictive abilities, respectively. In our study, high values of R^2^Y and Q^2^Y in these PLS models were displayed. For example, in striatum, R^2^Y was 0.985 and Q^2^Y was 0.974, which forcefully indicated that OSC-PLS model in our study had good or excellent fitness and predictive abilities, thus being valid (Figure 3).

### Metabolic changes in mice hippocampus, NAc, PFC and striatum

**Figure 4:**
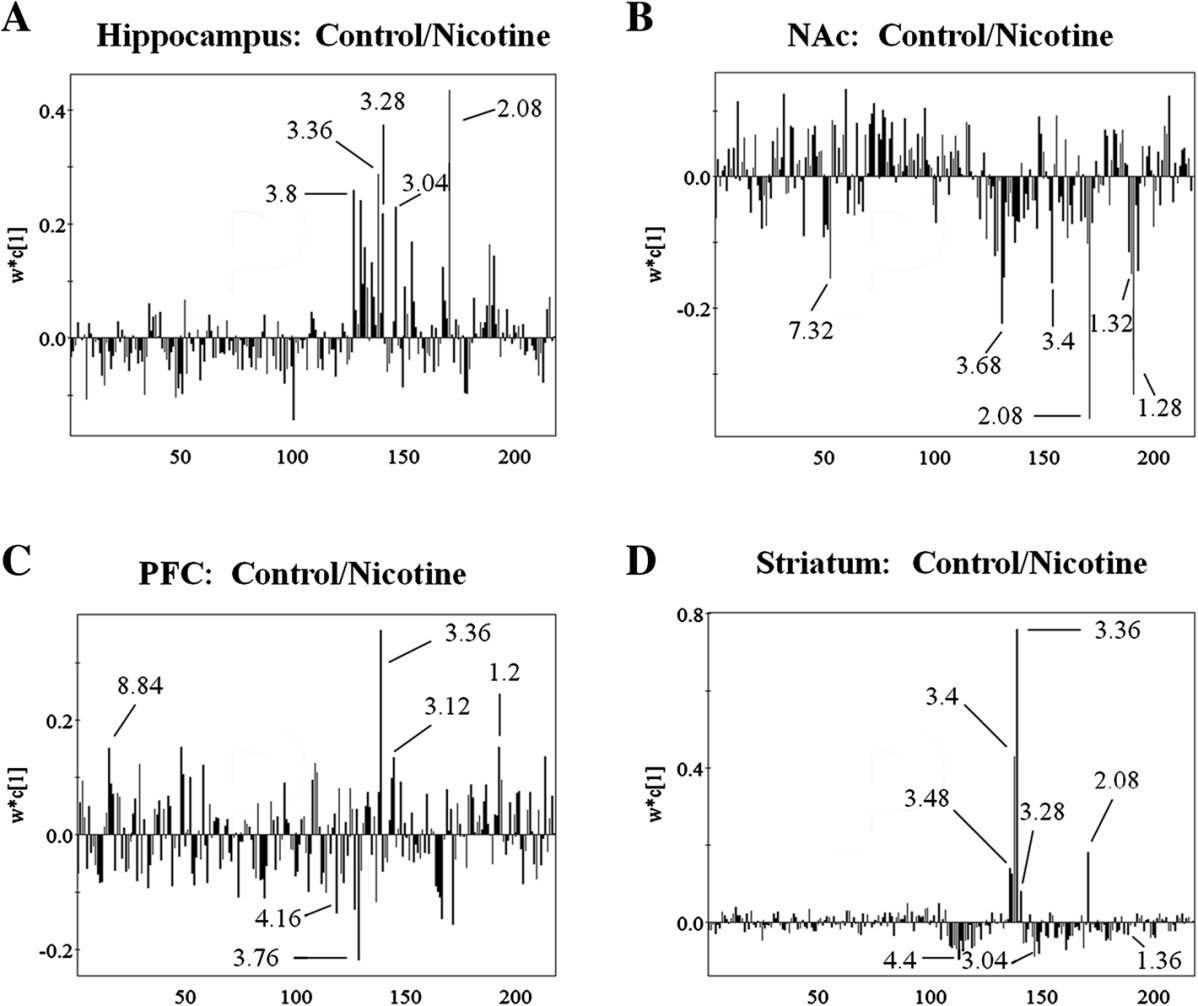
Comparison of brain region from mice between control group and nicotine group by applying PLS loading plots for the region 9.4–5.1 and 4.6–0.2 ppm (218 segments) after application of OSC data filter; (A) hippocampus in control group and nicotine group; (B) NAc in control group and nicotine group; (C) PFC in control group and nicotine group; (D) striatum in control group and nicotine group.

To investigate metabolic changes of brain hippocampus, NAc, PFC and striatum in control and nicotine groups, PLS loading plots after application of OSC data filter was applied to analyze ^1^H NMR data (Figure 4). In hippocampus, the metabolites that predominantly contributed to the separation of two groups were glutamate, creatine, glutathione, taurine, glucose and 1-methylhistidine (Figure 4A and Table 1). For example, the 2.08 bucket indicated lower glutamate from hippocampus of nicotine group than control (Figure 4A). Moreover, the 3.4 bucket indicated higher glucose from NAc (Figure 4B and Table 2), but lower glucose from PFC in nicotine-treated mice (Figure 4C and Table 3). In striatum, creatine (3.04 ppm) of nicotine group showed higher level than that of control (Figure 4D and Table 4). Correspondingly, according to the VIP values (VIP ≥ 1) in the OSC-PLS models, the differentially changed metabolites in the hippocampus, NAc, PFC and striatum of mice were identified and listed in Tables 1– 4, respectively.

**Table 1:** Summary of the variations from hippocampus metabolites in mice

**Metabolites**	**Chemical shift**	**Control-nicotine**	
	**(ppm)**	**VIP**^**a**^	**Loading**^**b**^
Glutamate	2.08	6.07	-0.433
Cystine	3.36	4.64	-0.287
Creatine	3.04	3.88	-0.229
Glutathione	3.8	3.37	-0.258
1-Methylhistamine	3.68	3.28	-0.241
Taurine	3.28	2.79	-0.2 18
2-Ketobutyric acid	2.76	2.71	-0.167
*	3.6	2.19	-0.159
Glucose	3.40	2.11	-0.021
Lysine	1.36	2.09	-0.163

^a^Variable importance in the projection (VIP ≥ 1) was obtained from OSC-PLS.

^b^Loading: the negative (-) value represents a decrease of the metabolite in the second group of the subtitle (e.g.: Control → Nicotine).

The symbol (*) represents a material which has not been identified.

**Table 2:** Summary of the variations from NAc metabolites in mice

**Metabolites**	**Chemical shift**	**Control-nicotine**	
	**(ppm)**	**VIP**^**a**^	**Loading**^**b**^
Glutamate	2.08	5.02	+0.369
Isoleucine	1.28	4.46	+0.331
1 -Methyihistamine	3.68	2.99	+0.223
Cystine	3.36	2.96	+0.068
2-Ketobutyric acid	2.76	2.24	+0.162
Lactate	1.32	2.11	+0.148
Tryptamine	7.32	2.09	+0.155
Glycerol	3.64	2.04	+0.153
Glucose	3.40	1.92	+0.066
3 -Hydroxybutyric acid	1.20	1.90	+0.142

^a^Variable importance in the projection (VIP ≥ 1) was obtained from OSC-PLS.

^b^Loading: the positive (+) value represents an increase of the metabolite in the second group of the subtitle (e.g.: Control → Nicotine).

**Table 3:** Summary of the variations from PFC metabolites in mice

**Metabolites**	**Chemical shift**	**Control-nicotine**	
	**(ppm)**	**VIP**^**a**^	**Loading**^**b**^
Cystine	3.36	5.83	-0.354
Arginine	3.76	2.90	+0.217
Glucose	3.40	2.52	-0.074
Glutamate	2.04	2.06	+0.156
3-Hydroxybutyric acid	1.20	2.05	-0.151
Uracil	7.52	2.03	-0.151
NAD	8.84	1.99	-0.150
Acetone	2.24	1.92	+0.145
Phosphocholine	4.16	1.83	+0.135
L-Phenvlalanine	3.12	1.80	-0.133

^a^Variable importance in the projection (VIP ≥ 1) was obtained from OSC-PLS.

^b^Loading: the positive (+) value represents an increase of the metabolite in the second group of the subtitle (e.g.: Control → Nicotine), while the negative (-) value represents a decrease of the metabolite in the second group of the subtitle.

**Table 4:** Summary of the variations from striatum metabolites in mice

**Metabolites**	**Chemical shift**	**Control-nicotine**	
	**(ppm)**	**VIP**^**a**^	**Loading**^**b**^
Cystine	3.36	10.51	-0.76
Glucose	3.40	5.97	-0.43
Glutamate	2.08	3.37	-0.18
Acetoacetic acid	3.44	2.02	-0.124
Tryptophan	3.48	1.92	-0.138
Lysine	1.36	1.91	±0.03
Taurine	3.28	1.54	-0.08
Creatine	3.04	1.49	±0.088
Acetylcholine	3.24	1.36	±0.054
NAA	4.4	1.36	±0.095

^a^Variable importance in the projection (VIP ≥ 1) was obtained from OSC-PLS.

^b^Loading: the positive (+) value represents an increase of the metabolite in the second group of the subtitle (e.g.: Control → Nicotine), while the negative(-) value represents a decrease of the metabolite in the second group of the subtitle.

### Metabolites changes in neurotransmitters

Compared with saline control, glutamate, tryptamine and acetylcholine were significant altered in hippocampus, NAc and striatum in nicotine-treated mice. Glutamate (2.08 ppm), a neurotransmitter, was reduced in hippocampus and striatum but increased in NAc and PFC. Tryptamine (7.32 ppm), a precursor of 5-HT, was also increased in NAc. Moreover, acetylcholine (3.24 ppm) was elevated in striatum. The initial disturbances of neurotransmitters are listed in Table 2 to Table 4, respectively.

### Metabolites alterations in oxidative stress

In nicotine-treated mice, glutathione (3.8 ppm), an antioxidant, was decreased in hippocampus (Table 1);moreover, taurine (3.28 ppm), acting as a free radical scavenger and possessing cytoprotective properties, was significantly reduced in hippocampus and striatum(Table 4).

### Membrane disruption and mitochondria dysregulation caused by metabolites changes

Phosphocholine (4.16 ppm) and glycerol (3.64 ppm), membrane ingredients, were markedly increased by nicotine CPP paradigm, the former in PFC, the latter in NAc (Tables 2 and 3). Moreover, NAA (4.4 ppm), a marker of neuronal viability and metabolite synthesized in mitochondria, was increased distinctively in striatum (Table 4).

### Metabolites variations related to energy metabolism

Several metabolites, including glucose, lactate, creatine, acetone, 3-hydroxybutyrate and nicotinamide-adenine dinucleotide (NAD), were significantly modified in certain brain regions of nicotine-treated mice (Tables 1– 4). For example, glucose (3.4 ppm) was markedly decreased in hippocampus, PFC and striatum, but increased in NAc. Creatine (3.04 ppm), which stores energy for the cell by means of a phosphate covalent bond in a similar manner to ATP/ADP, was decreased markedly in hippocampus but increased in striatum. It is known that lactate provides an alternate energy source for brain. After nicotine CPP paradigm, the increases in lactate (1.32 ppm) and 3-hydroxybutyrate were found in NAc. However, acetone (2.24 ppm), 3-hydroxybutyrate (1.2 ppm) and NAD (8.84 ppm) were reduced in PFC.

### Metabolites alterations associated with amino acid

The levels of several amino acids were altered in the four brain regions of nicotine-treated mice, including glutamic acid, lysine, isoleucine, phenylalanine, arginine and cystine (Tables 1 and 4). Among these modified amino acids, cystine (3.36 ppm) was significantly dropped in hippocampus, PFC and striatum, but dramatically raised in NAc. Lysine (1.36 ppm) displayed a decrease in hippocampus; however, lysine in striatum was increased. Phenylalanine (3.12 ppm) in PFC and isoleucine (1.28 ppm) in NAc were reduced by nicotine. Moreover, a reduction of uracil (7.52 ppm) in PFC was obvious. These findings imply that nicotine causes disruptions of amino acid metabolisms in brain.

## Discussion

Many studies have been performed to explore the mechanisms underlying nicotine-induced rewarding effects, but molecular mechanisms still remain unclear. Metabonomics not only is involved within the system biology framework, but also provides a promising opportunity to explore the molecular mechanisms of diseases [24,25]. The current experiment was designed to study the metabolic profling of nicotine-induced CPP mice by applying ^1^H NMR-based metabonomic in four brain regions including hippocampus, PFC, NAc and striatum. Interestingly, we found that certain metabolites were markedly altered by nicotine. The modified metabolites are correlated with neurotransmitter disturbance, oxidative stress alteration, mitochondria dysregulation, membrane disruption, energy metabolism imbalance as well as amino acid disorder. We consider that the rewarding effect of nicotine in mice may be closely associated with these modified metabolites. In other words, changes in CPP behavior may contribute to differences between controls and nicotine-exposed animals in the metabolomic analysis.

### Disturbance in neurotransmitters

Nicotine, a biologically active substance, has widespread pharmacological properties in the central and peripheral nervous systems. It has been shown that nicotine regulates release of multiple neurotransmitters, including dopamine, norepinephrine, serotonin, acetylcholine and amino acids in various regions of rat brain such as the cortex, hippocampus, NAc, striatum [26,27]. Repeated administration of nicotine induces both behavioral sensitization and alterations in dopamine and glutamate transmission as well as glutamatergic synaptic plasticity [28,29]. Considerable researches into the neurobiology of cocaine addiction have displayed that glutamate plays a crucial role in both the initiation and expression of addiction related behaviors [30,31]. In nicotine-induced CPP mice, glutamate showed a low level in hippocampus and striatum, but displayed a high level in NAc and PFC. Considering that glutamate not only affects glutamatergic synaptic plasticity but also modulates initiation and expression of addiction behaviors, we infer that altered glutamate equilibrium in neurotransmitter signaling may participate in nicotine-induced CPP in mice [32].

Acetylcholine, an endogenous agonist of nAChR, is thought to modulate learning and memory by altering the oscillatory rhythms that result from the interaction of various hippocampal subregions [33,34]. In our study, nicotine enhanced acetylcholine level in striatum; moreover, an increase of tryptamine, a precursor of 5-HT, was displayed in NAc. Several preclinical studies suggest that acetylcholine exerts a myriad of effects on the addictive process and that persistent changes to the acetylcholine system induced by chronic drug use may enhance the risk of relapse. We speculate that nicotine can modify synaptic plasticity through altering neurotransmitter release, such as glutamate, acetylcholine and tryptamine.

### Oxidative stress

It is reported that nicotine is closely bound up with oxidative stresss [35]. Glutathione, creatine and taurine, which are ubiquitous and important antioxidant, protect mitochondria against endogenous oxygen radicals [36]. In our present study, these substances in brain were significantly altered by nicotine.

Glutathione, one of the most abundant intracellular antioxidants in the brain, can modulate the activity of NMDARs [37]. In our study, glutathione in hippocampus displayed a low level in nicotine-induced CPP mice, implying the weakened antioxidative capability due to the decrease in glutathione.

Creatine is thought to have a multifaceted role in the brain. In addition to being involved in brain osmoregulation, it has recently been related with energy homeostasis and direct antioxidant effects [38,39]. We found that creatine was significantly decreased in hippocampus but increased in striatum by nicotine. As creatine can store energy for the cell, the alterations of creatine in hippocampus and striatum suggest an energetic shift in different brain regions.

Taurine, a neuroprotective amino acid, has been reported to be a neuroprotective agent in numerous investigations [40]. It acts as a free radical scavenger, possesses cytoprotective abilities and prevents the damage from oxidative stress and apoptosis induced by toxicants in various cells and tissues [41]. In our study, taurine in hippocampus and striatum showed a remarkable decrease after nicotine treatment, indicating the disturbances of anti-oxidative stress in brain.

Taken above, we speculate that nicotine affects brain antioxidative and energy storage capacity by modulating glutathione, taurine and creatine levels. The central nervous system may take adaptive measures to prevent the damage from oxidation by consuming taurine and glutathione, thus resulting in stressful declines of glutathione and taurine in hippocampus and striatum.

### Membrane disruption and mitochondria dysregulation

It has been known that glycerol, phosphocholine and myo-inositol, acting as precursors for the synthesis of membrane phospholipids in the cell, exert a critical part on lipid metabolism [42]. Glycerol has been applied to investigate membrane phospholipid degradation in brain homogenates after cerebral ischemia and seizures [43]. The damage of membrane results in release of phospholipids and choline compounds, which are the major head of phospholipids [44]. Interestingly, we found increases of glycerol in NAc and phosphocholine in PFC in nicotine-induced CPP mice. The elevated glycerol and phosphocholine may reflect an enhanced degradation of cellular membranes in brain, hinting the disruption in membrane transportation or barrier function.

NAA is regarded as a biomarker of neuronal viability. Numerous evidences have indicated that NAA may be linked with multiple roles in neurons, such as neuronal metabolic function in mitochondria, myelinogenesis, osmoregulation [45]. Depletion of NAA is considered to be a reflection of neuronal loss or dysfunction [46]. In our present study, NAA in striatum showed an increased level in nicotine-induced CPP mice, suggesting that nicotine may be related to neuronal activity and modulate mitochondria function [47,48].

### Energy metabolism

The brain regulates energy homeostasis by balancing energy intake, expenditure and storage [49]. Neuronal activity is extremely energy demanding [50]. In our study, several metabolites related to energy metabolism were markedly modified by nicotine, including glucose, Lac, creatine, glycine and a-ketoglutaric acid.

NAD has been considered to be a key regulator of metabolism, stress resistance and longevity [51]. Moreover, NAD and NADH are the components of the central redox pair within cells and substrates of many dehydrogenases involved in brain energy metabolism [52]. In nicotine-induce CPP mice, NAD displayed a significant reduction in PFC. Addiction is a progress of energy consumption, so the decreased NAD in brain may reflect an enhanced energy demand induced by nicotine.

Glucose, a primary energy substrate for brain metabolism, plays an important role in energy homeostasis [53-55]. We found that glucose was decreased in striatum, hippocampus and PFC,but increased in NAc by nicotine CPP paradigm. Nicotine stimulates brain metabolism, which leads to a significant increase in glucose transporter densities and local cerebral glucose utilization; moreover, nicotine can effectively improve learning and cognitive functions [56,57]. It is reported that cognition is highly energy dependent and glucose acts as main energy substrate of neurons [58]. Thinking above, our findings suggest that nicotine could modulate energy metabolism in various brain regions.

Ketone bodies (D-3-hydroxybutyrate (3OHB) and acetoacetate) serve as an energy source alternative to glucose for the brain and enter the blood brain barrier by the monocarboxylate transporter 1 [59]. The proliferation and metabolic activity of neuroglial cells can be significantly enhanced by 3-HB and derivatives [60]. In our study, 3-HB and acetone were significantly decreased in PFC, but 3-HB was increased in NAc in nicotine-treated mice.

It is known that memory formation can increase synaptic transmission and morphological alterations at the synapse, both of which consume more energy in the neuron [61,62]. Taken above, it is reasonable to infer that nicotine-induced changes in these metabolites may reflect an energetic shift and alteration of energy storage capacity.

### Changes in amino acid metabolism

Amino acid metabolism is extremely complex because large numbers of metabolites are involved. The disorder of amino acid metabolism is probably induced by gluconeogenesis, proteolysis and oxidative catabolism [63]. Amino acids as substrates are extremely equired for energy production during infection [64]. It is reported that a certain of metabolites, including sarcosine, uracil, kynurenine, glycerol-3-phosphate, leucine and proline, display a high level in metastatic prostate cancer and can potentially be regarded as biomarkers for progressive disease [65]. Similarly, we also found that several amino acids were significantly altered, including cystine, arginine, isoleucine, glutamate, lysine and phenylalanine. Because these amino acids belong to essential amino acids, non-essential amino acids or amino acid with putative neurotransmitter function, we speculate that protein metabolism in brain could be influenced by nicotine due to the alteration of amino acids.

## Conclusion

This study developed nicotine CPP paradigm in C57BL/6 J mice and applied metabolomics based on ^1^H NMR spectroscopy to explore the metabonomic profiling in four brain regions. We found that several metabolites were specifically modified by nicotine-induced CPP mice. The modified metabolites include neurotransmitter, energy source, membrane components and amino acids, which may be the outcome of the adaptive measures taken by brain in response to nicotine stimulation. Our results show that nicotine CPP may contribute to a certain region-specific changes of metabolites and that global metabolite profiling can provide a new insight into the mechanism about rewarding effect of nicotine.

## Abbreviations

NAc: Nucleus acumens; PFC: Prefrontal cortex; NAD: Nicotinamide-adenine dinucleotide; VTA: Ventral tegmental area; NMR: Nuclear magnetic resonance; CPP: Conditioned place preference; PCA: Coupled with principal component analysis; PLS: Partial least squares; OSC: Orthogonal signal correction; ANOVA: One-way analysis of variance; CPMG: Carr-Purcell-Meiboom-Gill; FIDs: Free induction decays; PLS: Partial least squares; PR: Pattern recognition; PCA: Principal component analysis; OSC: Orthogonal signal correction; VIP: Variable importance plot; PCs: Principal components; NAD: Nicotinamide-adenine dinucleotide.

## Competing interests

The authors declare that they have no competing interests.

## Authors’ contributions

Authors XC, HL and QB designed the study and wrote the protocol. Author BC, XS, ZH, YD, CD and RZ conducted literature searches and provided summaries of previous research studies. Author YL, QZ, BZ, DF, YZ and JH conducted the statistical analysis. Author HL wrote the first draft of the manuscript and all authors contributed to and have approved the final manuscript.
